# Difference in one-year late lumen loss between high- and low-dose paclitaxel-coated balloons for femoropopliteal disease

**DOI:** 10.1007/s00380-024-02370-0

**Published:** 2024-02-16

**Authors:** Kenji Kodama, Yoshimitsu Soga, Yusuke Tomoi, Nobuaki Sakai, Kazuaki Imada, Tomonori Katsuki, Hiroyuki Tabata, Kenji Ando, Yoshihisa Nakagawa

**Affiliations:** 1 Department of Cardiology, Shiga University of Medical Science Hospital, Seta Tsukinowa-Cho, Otsu, Shiga 520-2192 Japan; 2 Kokura Memorial Hospital, Fukuoka, Japan

**Keywords:** Lower extremity artery disease, Endovascular therapy for femoropopliteal artery, Paclitaxel coated balloon, Late lumen loss, Late lumen enlargement

## Abstract

The objective of the study is to investigate the difference in 1-year late lumen loss (LLL) between the high- (IN.PACT Admiral) and low-dose (Lutonix) paclitaxel-coated balloon (PCB). Although a recent randomized clinical trial demonstrated no difference in efficacy endpoint between high- and low-dose PCB, it remains unclear whether high-dose PCB was superior to low-dose PCB in actual clinical practice. We enrolled 64 patients with 67 de novo femoropopliteal lesions who underwent PCB angioplasty at Kokura Memorial Hospital from May 2014 to March 2020 and subsequent follow-up angiography after 1 year. The primary endpoint was 1-year LLL, whereas the secondary endpoints were binary restenosis and clinically driven target lesion revascularization (CD-TLR) after 1 year. The high- and low-dose PCB groups had 45 and 22 lesions, respectively. Although the low-dose PCB group had higher rates of coronary artery disease, hemodialysis, and chronic limb-threatening ischemia than the high-dose PCB group, the latter had a longer lesion length and more lesions with a TASC classification C or D than the former. The high-dose PCB group had a significantly lower LLL than the low-dose PCB group (0.40 ± 1.05 vs. 1.19 ± 1.03 mm; *P* = 0.003, respectively). Moreover, the high-dose PCB group had significantly lower rates of binary restenosis at 1 year than the low-dose PCB group (22.2% vs. 50.0%; *P* = 0.02, respectively). Moreover, negative LLL was only observed in the high-dose PCB group (33.3% vs. 0%, *P* = 0.005). The high-dose PCB group had a significantly lower LLL than the low-dose PCB group.

## Introduction

Endovascular therapy (EVT) using a paclitaxel-coated balloon (PCB) for femoropopliteal lesions has been found to promote better late lumen loss (LLL), binary restenosis rates, and clinically driven target lesion revascularization (CD-TLR) rates compared to plain balloon angioplasty [1–4]. A study confirmed the superiority of PCB over plain balloon angioplasty in Japanese patients [5]. Moreover, a recent study comparing PCB and scaffold device suggested that there was a significantly less re-occlusion rate in the PCB group in treatment for chronic total occlusion (CTO) of the superficial femoral artery (SFA) [6]. Several PCBs with a number of paclitaxel dosages ranging from 2.0 to 3.5 µg/mm^2^ are currently available on the market. A meta-analysis showed no change in LLL at 6 months after EVT using PCB regardless of the paclitaxel dose coated on the platform balloon [7]. However, an experimental study on swine demonstrated that paclitaxel levels in the superficial femoral artery were sustained longer in the high-dose (3.0 µg/mm^2^) than in the low-dose (2.0 µg/mm^2^) PCB group [8]. Another meta-analysis showed that the risk for TLR was significantly lower in the high-dose (3.5 µg/mm^2^) than in the low-dose (2.0 µg/mm^2^) PCB group [9]. Recently, Steiner et al. performed a direct comparison of two PCBs with different paclitaxel dosages and exhibited comparable results in terms of primary patency and CD-TLR [10]. However, no study has yet directly compared PCBs in terms of LLL, which has been found to effectively describe the degree of neointimal proliferation of target lesions after EVT. Therefore, the current study sought to determine the difference in LLL after 1 year between high-dose PCB and low-dose PCB groups.

## Methods

### Study design

**Fig. 1:**
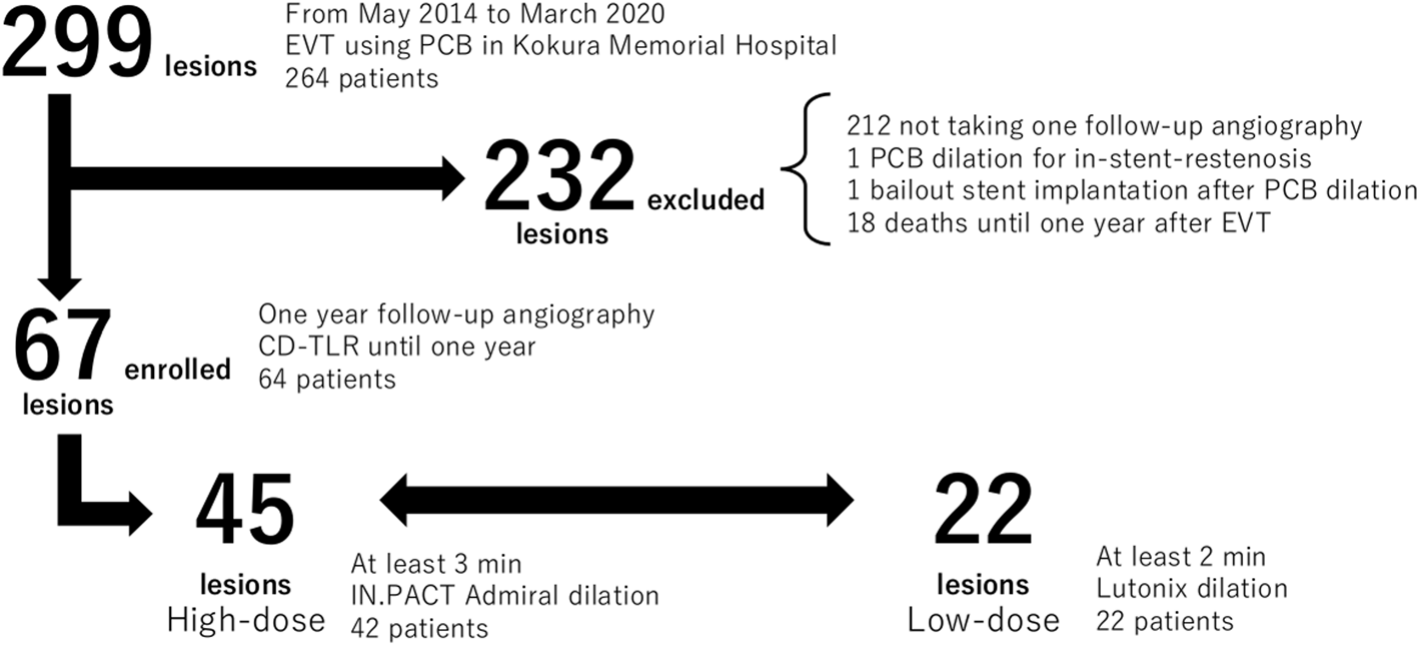
Flowchart for participant inclusion into this study. *EVT* endovascular therapy, *PCB* paclitaxel-coated balloon, *CD*-*TLR* clinically driven target lesion revascularization

This retrospective clinical study included patients who underwent EVT using PCB for femoropopliteal lesions at Kokura Memorial Hospital from May 2014 to March 2020. Patients eligible for the study received follow-up angiography 1 year after EVT or underwent CD-TLR until 1 year after EVT. Throughout the study period, 299 femoropopliteal lesions from 264 patients were treated using EVT with PCB. Among these lesions, 232 lesions were excluded owing to the absence of follow-up angiography 1 year after EVT (*n =* 212), EVT for in-stent-restenosis (ISR) (*n =* 1), bailout stent implantation (*n =* 1), and death until 1 year after EVT (*n =* 18). Therefore, 67 lesions were ultimately analyzed (Fig. 1). The study protocol was approved by the ethics committee at the hospital, and the study was conducted in accordance with the Declaration of Helsinki. We obtained consent from all patients using an opt-out procedure.

### Procedure

Two types of PCBs were dilated in the lesions: high-dose PCB (IN.PAC Admiral, Medtronic, Dublin, Ireland) and low-dose PCB (Lutonix, BARD, Tempe, Arizona). PCB inflation was maintained for > 3 min in cases receiving IN.PACT Admiral or >2 min in those receiving Lutonix. The selection of PCBs was at the physician’s discretion. All patients received dual antiplatelet therapy before the procedure. Two antiplatelet agents from aspirin (100 mg/day), clopidogrel (75 mg/day), or cilostazol (200 mg/day) were selected.

### Follow-up

Clinical follow-up was conducted 1 month, 6 months, and 1 year after the procedure. Follow-up angiography was performed after 1 year ± 3 months in the same direction after obtaining patient consent.

### Endpoints

The primary endpoint was LLL at 1 year after the procedure. LLL was defined as the minimum lumen diameter (MLD) immediately after EVT minus the MLD at the 1-year follow-up. LLL was assessed using subsegmental angiographic analysis. This method involves dividing each treated lesion into 10 equidistant subsegments. The minimal diameter within each subsegment was then measured immediately after the procedure and again during follow-up after 1 year by quantitative vessel angiography (QVA). The mean LLL at the 1-year follow-up was calculated [11]. QVA analyses were performed using the Cardiovascular Angiography Analysis System 5 Workstation and were calibrated using a ruler or the inner diameter of the catheter.

Secondary outcomes included binary restenosis and CD-TLR. Binary restenosis was defined as a peak systolic velocity ratio > 2.4 on duplex ultrasonography or > 50% stenosis on angiography. CD-TLR was defined as reintervention performed due to diameter stenosis of >50% in the target lesion and evidence of recurrent clinical symptoms.

### Statistical analysis

Continuous variables are presented as mean ± standard deviation, whereas categorical variables are presented as percentages. Differences among the groups were determined using Student’s *t* test for continuous variables and the chi-square or Fisher exact test for categorical variables. All statistical analyses were performed using JMP^®^ 16 (SAS Institute Inc., Cary, NC, USA), with *P* < 0.05 indicating statistical significance.

## Results

### Baseline (Table 1)

**Table 1:** Baseline Characteristics

	High-dose PCB (*N* = 42)	Low-dose PCB (*N* = 22)	*P* value
Age (yrs)	74.4 ± 7.9	73.6 ± 8.1	0.70
Male (%)	26 (61.9)	15 (68.2)	0.62
Current smoker (%)	5 (11.9)	6 (27.3)	0.12
Hypertension (%)	35 (83.3)	19 (86.4)	0.75
Diabetes mellitus (%)	24 (57.1)	16(72.7)	0.22
Dyslipidemia (%)	29 (69.1)	18 (81.8)	0.27
Renal insufficiency^a^ (%)	21 (50.0)	13 (59.1)	0.49
Regular dialysis (%)	8 (19.1)	10 (45.5)	0.02
Coronary artery disease (%)	26 (61.9)	19 (86.4)	0.04
Cerebrovascular disease (%)	13 (31.0)	3 (13.6)	0.12
CLTI (%)	3 (7.1)	7 (31.8)	0.01
Rutherford category			
2/3/4/5	25/17/1/2	9/6/0/7	0.02
Pre-procedural ABI	0.70 ± 0.20	0.73 ± 0.16	0.64
Medication			
Aspirin (%)	29 (69.1)	18 (81.8)	0.27
Clopidogrel (%)	38 (90.5)	19 (86.4)	0.62
Cilostazol	8 (19.1)	5 (22.7)	0.73
Anticoagulant (%)	7 (16.7)	4 (18.2)	0.87
Lipid-lowering drugs (%)	35 (77.8)	16 (72.7)	0.65
Lesion	(N = 45)	(N = 22)	
SFA (%)	39 (86.7)	16 (72.7)	0.16
Pop A (%)	6 (13.3)	6 (27.3)	0.16
Lesion Length (mm)	158.3 ± 67.2	118.3 ± 67.6	0.03
Reference diameter (mm)	5.17 ± 0.75	5.21 ± 0.62	0.81
Chronic total occlusion (%)	14 (31.1)	4 (18.2)	0.26
TASC classification			
A/B/C/D	3/22/15/5	10/8/3/1	0.002
PACCS grade			
0/1/2/3/4	14/13/8/3/7	3/5/5/1/8	0.27
No. of patent runoff vessels			
0/1/2/3	4/10/24/7	3/8/7/4	0.39
Procedual characteristics			
Predilation (%)	41 (91.1)	22 (100.0)	0.16
Approach (%)			
Ipsilateral/contralateral	1 (2)/44 (98)	8 (36)/14 (64)	0.0001
Sheath size			
5F/6F	3 (7)/42 (93)	12 (55)/10 (45)	< 0.0001
Dissections post-procedure			
None/A/B/C/D/E/F	8/11/21/4/1/0/0	7/7/7/1/0/0/0	0.62
Diameter stenosis post-procedure (%)	37.0 ± 11.8	37.1 ± 10.7	0.98

Data are shown as mean ± SD or n (%)

ABI, ankle-brachial index; CLTI, chronic limb-threatening ischemia; SFA, superficial femoral artery; Pop A, popliteal artery

^a^Defined as estimated glomerular filtration rate < 60 ml/min/1.73 m^2^

The current study analyzed 67 lesions (64 patients). The high-dose PCB group included 45 lesions (42 patients) and the low-dose PCB group included 22 lesions (22 patients), respectively. Patient and lesion characteristics are summarized in Table 1. The rates of coronary artery disease, hemodialysis, and chronic limb-threatening ischemia (CLTI) were significantly higher in the low-dose PCB group than in the high-dose PCB group. Regarding lesion characteristics, the high-dose PCB group had a significantly longer lesion length and more lesions with a TASC classification of C or D compared to the low-dose PCB group, although no significant differences were found in terms of PACCS grade, number of patent infrapopliteal runoff vessels, and dissection grade after the procedure.

### Primary outcome (Fig. 2 and Table 2)

**Fig. 2:**
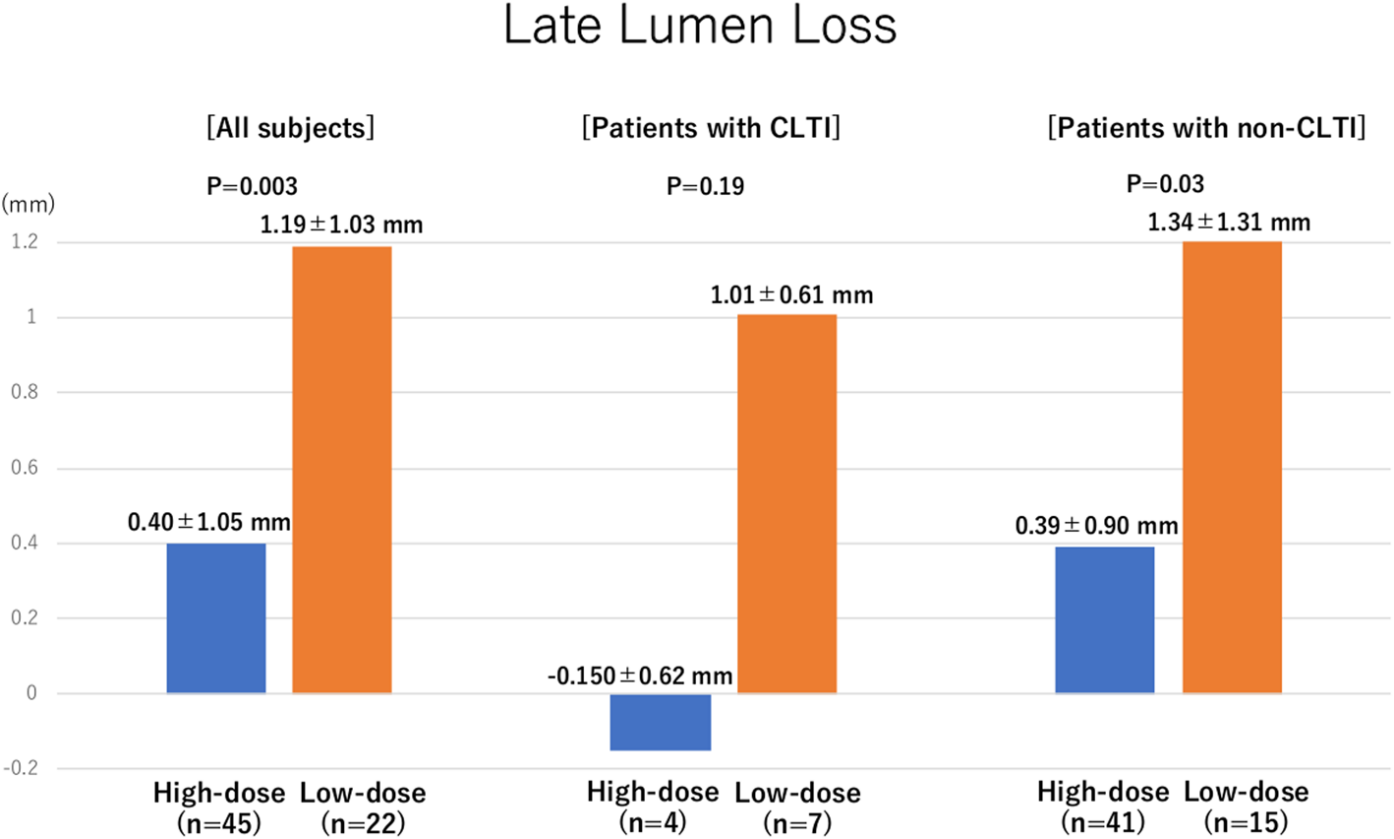
The result of the primary outcome. The late lumen losses in the groups of all subjects and patients with CLTI were assessed, respectively. *CLTI* chronic limb-threatening ischemia

**Table 2:** Analysis of quantitative vascular angiography

	High-dose-PCB (*N* = 45)	Low-dose-PCB (*N* = 22)	*P* value
Pre-procedure			
Lesion length (mm)	158.3 ± 67.2	118.3 ± 67.6	0.02
Reference vessel diameter (mm)	5.17 ± 0.75	5.21 ± 0.62	0.78
Post-procedure			
MLD (mm)	4.20 ± 0.66	4.25 ± 0.72	0.78
One-year follow-up			
MLD (mm)	3.88 ± 1.19	3.07 ± 0.93	0.007
Negative LLL (%)	15 (33.3)	0 (0.0)	0.002
CLTI group	*N* = 4	*N* = 7	
Post-procedural MLD (mm)	4.51 ± 0.69	4.02 ± 0.87	0.39
One-year follow-up MLD (mm)	4.66 ± 0.73	3.01 ± 0.54	0.04
Non-CLTI group	*N* = 41	*N* = 15	
Post-procedural MLD (mm)	4.18 ± 0.68	4.47 ± 0.64	0.19
One-year follow-up MLD (mm)	3.79 ± 1.08	3.12 ± 1.19	0.09

Data are shown as mean ± SD or n (%)

*MLD* minimum lumen diameter; CLTI, chronic limb-threatening ischemia, *MLD* was assessed using subsegmental angiographic analysis

**Fig. 3:**
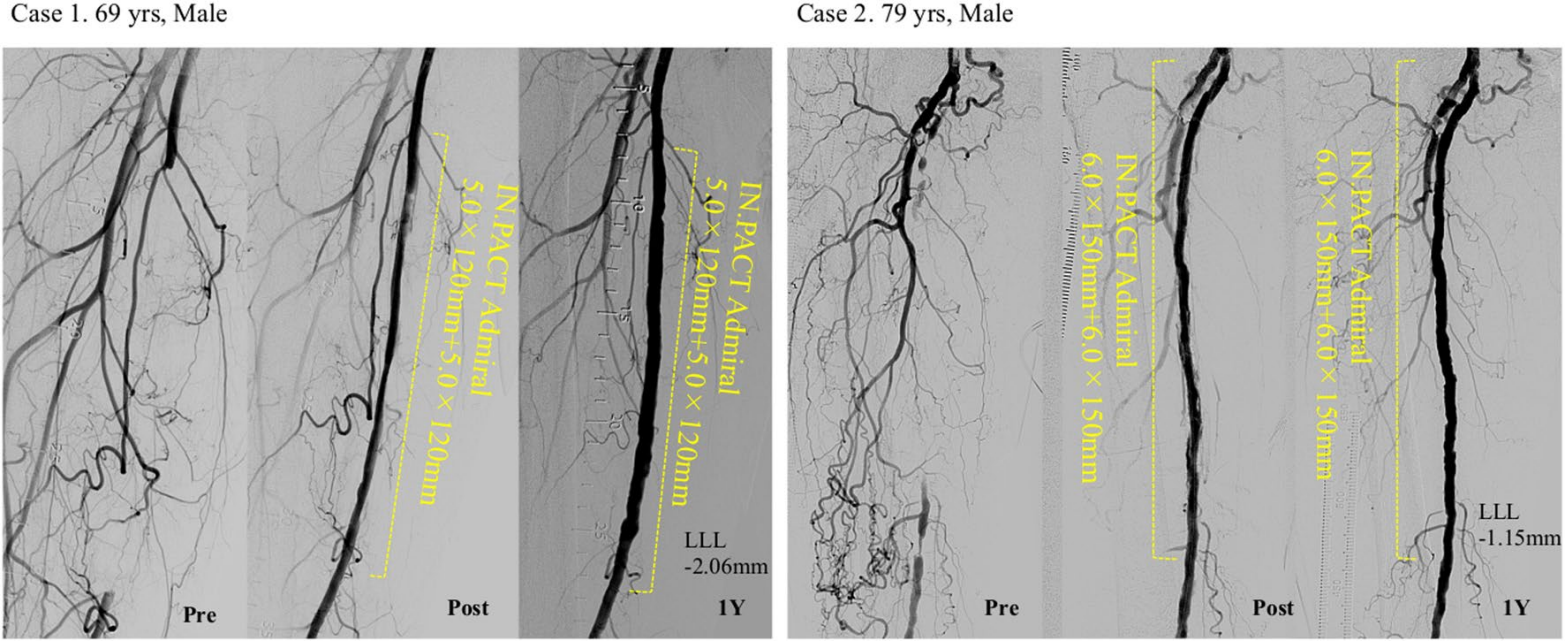
Representative cases of negative late lumen loss. *LLL* late lumen loss

The high-dose PCB group had a significantly lower LLL than the low-dose PCB group (0.40 ± 1.05 vs. 1.19 ± 1.03 mm; *P* = 0.003, respectively). Although the difference in LLLs between both groups was not significant among those with CLTI, the high-dose PCB group had lower LLL compared to the low-dose PCB group (− 0.15 ± 0.62 vs. 1.01 ± 0.61 mm; *P* = 0.19). Among the cases in the high-dose PCB group, 15 (33.3%) had negative lumen loss after 1 year, whereas none of the cases in the low-dose PCB group had the same (Table 2). The representative cases are presented in Fig. 3.

### Secondary outcome (Table 3)

**Table 3:** Rates of binary restenosis and CD-TLR

	High-dose PCB	Low-dose PCB	*P* value
Binary restenosis^a^			
All	10/45 (22.2)	11/22 (50.0)	0.02
CLTI group	0/4 (0.0)	4/7 (57.1)	0.04
Non-CLTI group	10/41 (24.4)	7/15 (46.7)	0.10
CD-TLR^b^			
All	9/45 (20.0)	9/22 (40.9)	0.17
CLTI group	0/4 (0.0)	2/7 (28.6)	0.47
Non-CLTI group	9/41 (22.0)	7/15 (46.7)	0.06

Data are shown as *n* (%)

^a^Defined as a peak systolic velocity ratio > 2.4 by duplex ultrasound scan or > 50% stenosis by angiography

^b^Defined as the reintervention performed due to greater than 50% diameter stenosis of the target lesion and evidence of recurrent clinical symptoms

After 1 year, the high-dose PCB group had a significantly lower binary restenosis rate (22.2% vs. 50.0%; *P* = 0.02, respectively) and CD-TLR rate (20.0% vs. 40.9%; *P* = 0.07, respectively) compared to the low-dose group, although the difference was not significant. Similar results were also found among patients with CLTI although the differences in these outcomes were not significant.

## Discussion

The current study directly compared two PCBs with different paclitaxel dosages in terms of LLL at 1 year. The main findings of the present study were as follows: (1) the high-dose PCB group had significantly lower LLL than the low-dose PCB group and (2) negative lumen loss was only observed in the high-dose PCB group.

The present study utilized subsegmental angiographic analysis to assess LLL after 1 year. Classic angiographic assessments are based on a single MLD measurement within the entire target lesion. However, this method may not depict the true LLL in cases wherein lesion morphology is not uniform along the entire lesion and relocation of the MLD site occurs during follow-up angiography after 1 year [10]. As such, the subsegmental angiographic measurement may provide a more comprehensive representation of the entire target lesion. The high-dose PCB group demonstrated better effectiveness than the low-dose PCB group given that their lower LLL was based on subsegmental angiographic analysis. Moreover, the high-dose PCB group also had significantly lower rates of binary restenosis than the low-dose PCB group. However, the low-dose PCB group had significantly more patients with CLTI than the high-dose group. EVTs for patients with CLTI were often performed via the ipsilateral approach using a 5-Fr guiding catheter considering that most of them had lesions occurring below the knee. Lutonix was compatible with a 5-Fr guiding catheter, which had a diameter of 5 or 6 mm and was often used for femoropopliteal lesions. On the other hand, IN.PACT Admiral was compatible with a 6-Fr guiding catheter. This was thought to explain why more patients with CLTI were included in the low-dose PCB group. Actually, EVTs for patients in the low-dose PCB group were more significantly often performed via the ipsilateral approach using a 5-Fr guiding catheter compared with those in the high-dose PCB group. However, the high-dose PCB group also had lower LLL and rates of binary restenosis and CD-TLR compared to the low-dose PCB group among patients with CLTI although the differences were not significant. Two meta-analyses demonstrated that high-dose PCB was superior to low-dose PCB in terms of efficacy outcome [6, 8]. However, the COMPARE study suggested that two different PCB doses exhibited comparable results in terms of primary patency and CD-TLR [9]. Using Lutonix as the low-dose PCB might have affected the results of this study which demonstrated the superiority of high-dose PCB. An experimental study in rabbits demonstrated that after PCB dilation in the infrarenal aorta, plasma paclitaxel concentrations were significantly higher for Lutonix than for the other four PCBs, including IN.PACT Admiral and RANGER, whereas paclitaxel concentrations in the aorta were significantly lower for Lutonix than the other PCBs [12]. Moreover, a multicenter, prospective study that observed the 1-year outcomes of PCB treatment for femoropopliteal lesions demonstrated that 1-year risk of restenosis was independently associated with Lutonix use [13]. In Japan, the clinical results for RANGER remain insufficient given that it had just been recently available. Therefore, future studies involving RANGER are certainly warranted.

Negative lumen losses were observed in 33.3% (15/45) of lesions in the high-dose PCB group but not in the low-dose PCB group. Several reports have shown that PCB treatment for the de novo coronary and peripheral arteries promoted late lumen enlargement [14–16]. In fact, Gongora et al. demonstrated that paclitaxel tissue levels after balloon inflation were comparable between the IN.PACT Admiral and Lutonix groups; however, the Lutonix group showed a sharp decrease in tissue levels 24 h after balloon dilation, whereas the IN.PACT Admiral group maintained increased higher tissue levels 7 days after the procedure [7]. Kobayashi et al. presented a case that exhibited paclitaxel retention on the intima of an SFA lesion using optical frequency domain imaging (OFDI) 42 days after EVT in an elderly woman using IN.PACT Admiral [17]. A recent report showed that more than half of cases with residual stenosis after PCB angioplasty for FP lesions might have stenosis regression 6 months after the procedure [18]. These data suggested that the high-dose PCB group had a longer inhibition of neointimal proliferation compared to the low-dose PCB group and that only the high-dose PCB group might have negative lumen loss.

## Limitations

The current study has several limitations worth noting. First, this study was a retrospective nonrandomized single-center study with a small sample size. Also, there were significantly different characteristics of patients and lesions among the two groups. The subgroup analysis of patients with CLTI was conducted because the sample size was not large enough to allow the multivariate analysis. Even after subgroup analysis, the high-dose -PCB group had more effective outcomes than the low-dose PCB group. Therefore, the result of this study was thought to be sufficiently significant. Second, there might exist several selection biases in this study, because the selection of PCBs and the regimen of follow-up angiography were at the physician’s discretion. To overcome these limitations, a prospective, randomized, multi-center study is warranted. Third, intravascular ultrasound, which would have allowed the accurate evaluation of LLL, was not used in follow-up angiography.

## Conclusion

LLL at 1 year was significantly lower in the high-dose than in the low-dose PCB group. Moreover, treatment for de novo femoropopliteal lesions using high-dose PCB could lead to late lumen enlargement. Further investigation should be needed to clarify the difference in effective outcomes between different doses of paclitaxel.

## References

[1] Tepe G, Laird J, Schneider P, Brodmann M, Krishnan P, Micari A, Metzger C, Scheinert D, Zeller T, Cohen DJ, Snead DB, Alexander B, Landini M, Jaff MR (2015). Drug-coated balloon versus standard percutaneous transluminal angioplasty for the treatment of superficial femoral and popliteal peripheral artery disease: 12-month results from the IN.PACT SFA randomized trial. Circulation.

[2] Rosenfield K, Jaff MR, White CJ, Rocha-Singh K, Mena-Hurtado C, Metzger DC, Brodmann M, Pilger E, Zeller T, Krishnan P, Gammon R, Müller-Hülsbeck S, Nehler MR, Benenati JF, Scheinert D (2015). Trial of a paclitaxel-coated balloon for femoropopliteal artery disease. N Engl J Med.

[3] Tepe G, Zeller T, Albrecht T, Heller S, Schwarzwälder U, Beregi JP, Claussen CD, Oldenburg A, Scheller B, Speck U (2008). Local delivery of paclitaxel to inhibit restenosis during angioplasty of the leg. N Engl J Med.

[4] Steiner S, Willfort-Ehringer A, Sievert H, Geist V, Lichtenberg M, Del Giudice C, Sauguet A, Diaz-Cartelle J, Marx C, Ströbel A, Schult I, Scheinert D (2018). 12-Month results from the first-in-human randomized study of the ranger paclitaxel-coated balloon for femoropopliteal treatment. JACC Cardiovasc Interv.

[5] Iida O, Soga Y, Urasawa K, Saito S, Jaff MR, Wang H, Ookubo H, Yokoi H (2018). Drug-coated balloon vs standard percutaneous transluminal angioplasty for the treatment of atherosclerotic lesions in the superficial femoral and proximal popliteal arteries: one-year results of the MDT-2113 SFA Japan randomized trial. J Endovasc Ther.

[6] Shimada T, Shima Y, Takahashi K, Miura K, Takamatsu M, Ikuta A, Habara S, Tanaka H, Goto T, Izumiya Y, Kadota K (2022). Delayed stenosis regression after drug-coated balloon angioplasty for femoropopliteal artery lesions. Heart Vessels.

[7] Katsanos K, Spiliopoulos S, Paraskevopoulos I, Diamantopoulos A, Karnabatidis D (2016). Systematic review and meta-analysis of randomized controlled trials of paclitaxel-coated balloon angioplasty in the femoropopliteal arteries: role of paclitaxel dose and bioavailability. J Endovasc Ther.

[8] Gongora CA, Shibuya M, Wessler JD, McGregor J, Tellez A, Cheng Y, Conditt GB, Kaluza GL, Granada JF (2015). Impact of paclitaxel dose on tissue pharmacokinetics and vascular healing: a comparative drug-coated balloon study in the familial hypercholesterolemic swine model of superficial femoral in-stent restenosis. JACC Cardiovasc Interv.

[9] Cassese S, Ndrepepa G, Fusaro M, Kufner S, Xhepa E (2019). Paclitaxel density and clinical efficacy of drug-coated balloon angioplasty for femoropopliteal artery disease: meta-analysis and adjusted indirect comparison of 20 randomised trials. EuroIntervention.

[10] Steiner S, Schmidt A, Zeller T, Tepe G, Thieme M, Maiwald L, Schröder H, Euringer W, Ulrich M, Brechtel K, Brucks S, Blessing E, Schuster J, Langhoff R, Schellong S, Weiss N, Scheinert D (2020). COMPARE: prospective, randomized, non-inferiority trial of high- vs. low-dose paclitaxel drug-coated balloons for femoropopliteal interventions. Eur Heart J.

[11] Liistro F, Weinberg I, Almonacid Popma A, Shishehbor MH, Deckers S, Micari A (2022). Paclitaxel-coated balloons versus percutaneous transluminal angioplasty for infrapopliteal chronic total occlusions: the IN.PACT BTK randomised trial. EuroIntervention.

[12] Boitet A, Grassin-Delyle S, Louedec L, Dupont S, Lamy E, Coggia M, Michel JB, Coscas R (2019). An experimental study of paclitaxel embolisation during drug coated balloon angioplasty. Eur J Vasc Endovasc Surg.

[13] Soga Y, Takahara M, Iida O, Tomoi Y, Kawasaki D, Tanaka A, Yamauchi Y, Tobita K, Kozuki A, Fujihara M, Ando K, Investigators P (2023). Vessel patency and associated factors of drug-coated balloon for femoropopliteal lesion. J Am Heart Assoc.

[14] Kleber FX, Schulz A, Waliszewski M, Hauschild T, Böhm M, Dietz U, Cremers B, Scheller B, Clever YP (2015). Local paclitaxel induces late lumen enlargement in coronary arteries after balloon angioplasty. Clin Res Cardiol.

[15] Scheller B, Fischer D, Clever YP, Kleber FX, Speck U, Böhm M, Cremers B (2013). Treatment of a coronary bifurcation lesion with drug-coated balloons: lumen enlargement and plaque modification after 6 months. Clin Res Cardiol.

[16] Shimada T, Habara S, Tanaka H, Kadota K (2021). Plaque volume reduction after drug-coated balloon angioplasty for superficial femoral artery lesion. Cardiovasc Interv Ther.

[17] Kobayashi N, Hirano K, Yamawaki M, Araki M, Sakai T, Sakamoto Y, Mori S, Tsutsumi M, Nauchi M, Sahara N, Honda Y, Makino K, Shirai S, Mizusawa M, Sugizaki Y, Nakano T, Fukagawa T, Kishida T, Kozai Y, Setonaga Y, Goda S, Ito Y (2020). Sustained drug retention after paclitaxel-coated balloon angioplasty for superficial femoral artery disease: follow-up intravascular imaging. SAGE Open Med Case Rep..

[18] Hayakawa N, Kodera S, Arakawa M, Hirano S, Shakya S, Kanda J (2022). Clinical outcome of drug-coated balloon versus scaffold device in patients with superficial femoral artery chronic total occlusion. Heart Vessels.

